# Underwater Target Perception in Local HOS Space

**DOI:** 10.1155/2021/5190655

**Published:** 2021-09-17

**Authors:** Jue Gao, Peiyi Zhu

**Affiliations:** School of Electrical Engineering and Automation, Changshu Institute of Technology, Changshu 215500, Jiangsu, China

## Abstract

In this paper, we propose an underwater target perception architecture, which adopts the three-stage processing including underwater scene acoustic imaging, local high-order statistics (HOS) space conversion, and region-of-interest (ROI) detection. After analysing the problem of the underwater targets represented by the acoustic images, the unique cube structure of the target in local skewness space is noticed, which is used as a clue to develop the ROI detection of underwater scenes. In order to restore the actual appearance of the ROI as much as possible, the focus processing is explored to achieve the target reconstruction. When the target size and number are unknown, using an uncertain theoretical template can achieve a better target reconstruction effect. The performance of the proposed method in terms of SNR, detection rate, and false alarm rate is verified by experiments with several acoustic image sequences. Moreover, target perception architecture is general and can be generalized to a wider range of underwater applications.

## 1. Introduction

Interpreting target information from acoustic images has long been an active research field [1] in ocean acoustics. According to the different application requirements [2–5], the research is mainly carried out along the direction of target detection, target recognition, target classification, and target tracking. An essential step is to search the region of interest (ROI), associated with the potential target, in the acoustic image. Conventional methods take local contrast as a cue to divide the acoustic image into different areas and mark specific areas as the ROI. Following this strategy, a variety of methods [6–8] including threshold segmentation method, clustering method, mathematical morphology method, and level set method have been developed. Alternative approaches have tended to focus on model-based detection [9] or supervised learning [10]. In many cases, acoustic images are difficult to interpret due to multiple artifacts, low signal-to-noise ratio (SNR), and inadequate resolution.

One solution is to construct a local background distribution model and take the discontinuous isolated singular as the clue to correlate ROI. Higher-order statistics (HOS) is considered as a possible local background transformation method because it has been proven to be sensitive to outliers [11] and is suitable for image processing. Jacovitti [12] introduced the application of HOS in image decomposition, blind deconvolution, coding, and pattern recognition. A group of scholars use HOS for image processing to solve line detection [13], sea mine classification [14], motion estimation [15], edge extraction [16], etc. Furthermore, research showed that statistics of small pixels in the neighbourhood are able to accomplish considerable differentiation. Sharma et al. [17] proposed an image representation method based on local HOS for texture classification and face analysis. The most enlightening research is proposed by Maussang [18], which employed local HOS to detect small deterministic regions surrounded by random noise in the synthetic aperture sonar (SAS) image.

**Figure 1:**
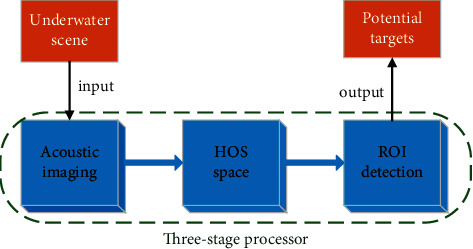
Underwater target perception architecture.

In this paper, we address the challenges of perceiving underwater targets in local HOS space of underwater scenes and, starting from our previous research [19, 20], we design a three-stage automatically processing architecture outlined in Figure 1. In the first stage, acoustic imaging is performed on the underwater scene in a certain field of view and mapped into corresponding acoustic images. In the second stage, the acoustic image is converted to the local HOS space, hoping to acquire clearer target information. In the third stage, the ROI detection algorithm is implemented in the local HOS space to remove abnormal target areas and screen out potential targets. We focus on two key challenges:

1. Can the local HOS space be a good representation of targets with low SNR in underwater scenes?
2. Can the target information obtained in the local HOS space be directly used for ROI detection?

The structure of the paper is organized as follows: Section 2 gives a brief review of the target representation in acoustic images and formulates the target representation in local HOS space.  Section 3 presents the ROI detection method in local HOS space and studies the target reconstruction by focus processing.  Section 4 provides experimental results of the proposed three-stage architecture on real acoustic image sequences and compares its performance with conventional methods. Finally, the conclusion is drawn in the last section.

## 2. Target Representation from Acoustic Images to Local HOS Space

### 2.1. Problem Formulation

**Figure 2:**
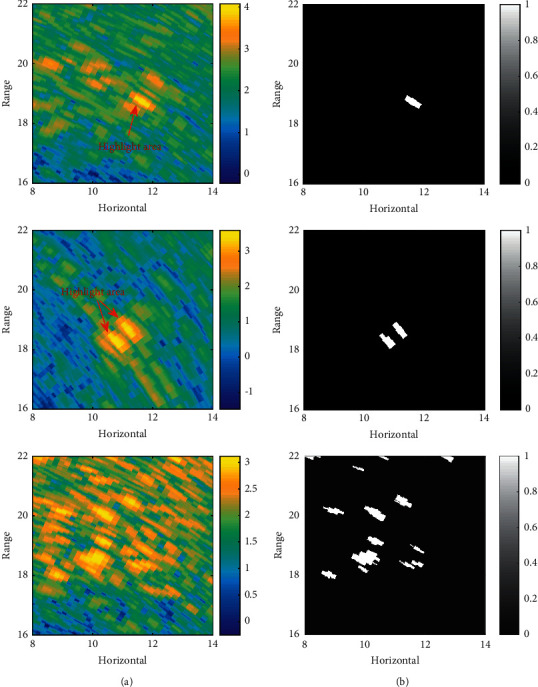
Target representations in different acoustic images: (a) targets in the acoustic image and (b) target segmentation.

A target in the underwater scene is usually represented as a set of specific pixels in acoustic images. The echo intensity, shape, and contour are used to describe this group of specific pixels, which become clues to indicate the presence or absence of the target in the image. Due to the complex and changeable characteristics of the underwater acoustic channel medium and its boundary, the highlight area associated with the potential target will also change significantly. Acoustic images of the same underwater scene collected at different time periods are shown in Figure 2(a), and the segmented highlighted area is shown in Figure 2(b). In the first line, the target appears as a long strip, which is a group of pixels whose echo intensity is higher than in the surrounding pixels. In the second line, there are two highlighted areas with similar shapes, indicating that the target is split into two subareas. In the third line, there are many highlight areas of different sizes, indicating that the target and noise reverberation have similar echo intensity, so it is difficult to distinguish which highlight areas correspond to potential targets.

The above analysis shows that the shape and contour features are not stable. In order to identify the target in the acoustic image sequence formed by the time-varying underwater scene, one solution is to explore the invariable local features [21, 22] and another solution is to establish a background model to indirectly identify underwater targets.

### 2.2. Target Representation in Local HOS Space

The background model of the acoustic image is established, which is transformed into HOS space, and the discontinuous distribution region is regarded as the potential target. According to previous research, Weibull distribution is used to represent the background of water imaging and skewness is chosen as the HOS. A rectangular window is designed to traverse the entire acoustic image, and the local skewness of each unit in the window *S*_w_ is calculated by

(1) $$\begin{array}{c} S_{W}=\frac{\left[\alpha \left(1-2\alpha \right)A^{3}-3\alpha \left(1-2\alpha \right)m_{B\left(1\right)}A^{2}-3\alpha \left(m_{B\left(2\right)}-2\left(1-\alpha \right)m_{B\left(1\right)}^{2}\right)A+\left(m_{B\left(3\right)}-3\left(1-\alpha \right)m_{B\left(1\right)}m_{B\left(2\right)}+2{\left(1-\alpha \right)}^{2}m_{B\left(1\right)}^{3}\right)\right]}{{\left(1-\alpha \right)}^{1/2}{\left(\alpha A^{2}-2\alpha m_{B\left(1\right)}A+m_{B\left(2\right)}-\left(1-\alpha \right)m_{B\left(1\right)}^{2}\right)}^{3/2}}, \end{array}$$

where *α* is the ratio of the target to the background in the window, *m*_*B*(*r*)_ is the *r*-th origin moment of the background, and A is the average echo intensity of the target. In calculation, A is commonly replaced by the SNR, which is defined as the average power ratio of the target and background echo intensity:

(2) $$\begin{array}{c} \text{SNR}=20\;\log_{10}\left(\frac{\left|A-\mu_{B}\right|}{\sigma_{B}}\right), \end{array}$$

where *μ*_*B*_ and *σ*_*B*_ are the mean value and mean square error of the background, respectively.

**Figure 3:**
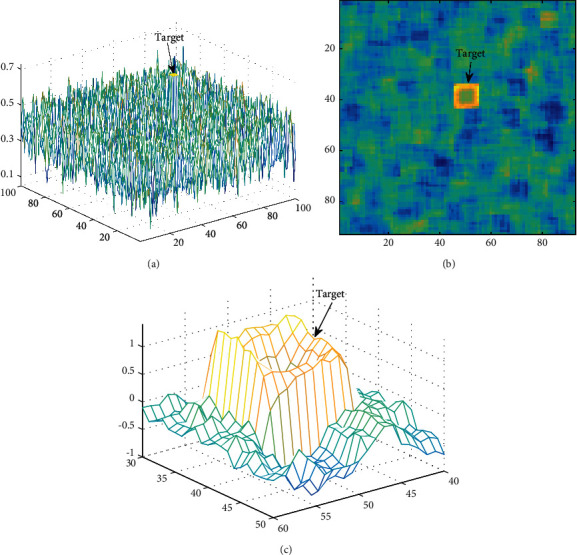
Local skewness for target representation (SNR=12 dB): (a) original acoustic image, (b) local skewness image, and (c) target details in local skewness space.

Local skewness can be regarded as a function of the target-to-background ratio and the SNR in the window. In the selection of sliding window size, a tradeoff must be made between obtaining high target contrast in the local skewness space and stable background distribution. A simulated acoustic image of 100 × 100 pixels was established, including a square target SNR = 12 dB with a side length *T*_o_ = 4, and the background followed Weibull distribution. Scale parameters *k*=5.2 and shape parameters *λ*=0.42 were estimated from the actual data. As seen in Figure 3(a), due to the small size of the target and weak SNR, the echo intensity is submerged in the surrounding background clutter. A sliding window of *T*_w_ = 7 is set to transform the original acoustic image to the local skewness space, and the deviation correction estimation of the local skewness is given by

(3) $$\begin{array}{c} {\hat{S}}_{W}=\frac{n^{2}}{\left(n-1\right)\left(n-2\right)}\frac{1/n\sum_{i=1}^{n}{\left(x_{i}-\bar{x}\right)}^{3}}{{\left[1/n-1\sum_{i=1}^{n}{\left(x_{i}-\bar{x}\right)}^{2}\right]}^{3/2}}, \end{array}$$

where *n* is the total number of pixels in the window.

In the local skewness space, the target displays a cube structure as shown in Figure 3(b), which can be easily identified from the background compared to the original acoustic image. As shown in Figure 3(c), the 3D representation of target details shows that the edge of this structure is high and the middle is low. The fewer the target pixels in the calculation window, the larger the *S*_W_, and as the target pixels increase in the calculation window, *S*_W_ gradually decreases.

## 3. ROI Detection and Target Reconstruction in HOS Space

### 3.1. ROI Detection

**Figure 4:**
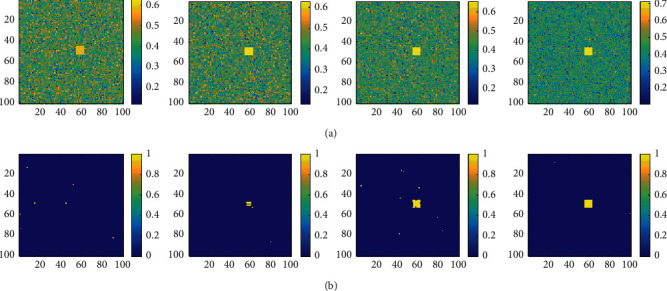
Detection on simulation images with lower SNR: (a) simulated acoustic image (6 dB, 8 dB, 10 dB, and 12 dB) and (b) corresponding detection results.

Once the SNR of the acoustic image is relatively low, the target will be submerged by the background, and it is difficult to identify the potential target directly by the detection algorithm. Create a 100 × 100 pixel simulated acoustic image containing a square target of size *T*_o_ = 8. Change the SNR to 6 dB, 8 dB, 10 dB, and 12 dB from left to right in Figure 4(a). The previously proposed subset censored-constant false alarm rate (SC-CFAR) algorithm [20] is used to detect the target in the above acoustic image, with *P*_fa_ = 0.01, and the detection result is shown in Figure 4(b). One can see that when the SNR is 6 dB, the target cannot be detected at all; When SNR is 8 dB or 10 dB, Only a part of the target can be detected. When the SNR reaches 12 dB, the target can be fully detected. The simulation results confirm that it is very difficult to detect the target in the acoustic image with lower SNR.

**Figure 5:**
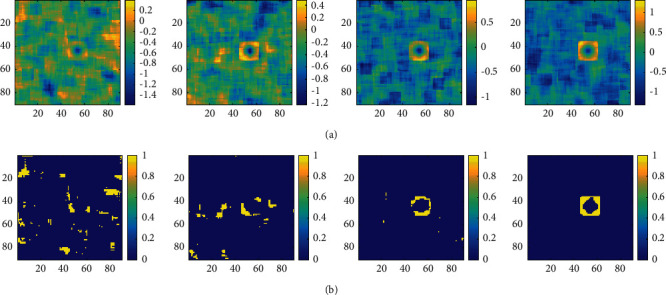
SC-CFAR detection on local skewness images: (a) local skewness image for Figure 4(a) and (b) corresponding detection results.

It is considered to implement detection in local HOS space through transformation. Let the window size *T*_w_ = 10; convert the original acoustic image in Figure 4(a) to the local skewness space, and the result is shown in Figure 5(a). It can be found that no matter whether the SNR of the original acoustic image is high or low, the target will form a unique cube structure in the local skewness image. The higher the SNR of the original acoustic image is, the clearer the target is and the easier it is to distinguish. Similarly, the SC-CFAR algorithm is executed in the local skewness space, and the detection result is shown in Figure 5(b). When the SNR is 6 dB and 8 dB, part of the cube structure can be detected but there are many false alarms. When the SNR is 10 dB, the number of false alarms is reduced but the cube structure is incomplete. When the SNR is 12 dB, the cube structure can be completely detected.

The simulation results show that the cube structure formed by the potential target in the local skewness space can be used as a clue of ROI, but the SC-CFAR algorithm will have a high false alarm rate in case of low SNR. In addition, the use of large computing windows will form a larger cube structure, which will cause problems in actual target positioning.

### 3.2. Target Reconstruction

The problem of ROI detection in the local HOS space mentioned above can be solved by target reconstruction. The focus processing is used to reconstruct the target for restoring the original appearance, and the local HOS image and target theoretical template are processed for correlation. The target theoretical template size *T*_M_ is related to the calculation window size *T*_w_ and the target size *T*_o_, and its expression is as follows:

(4) $$\begin{array}{c} T_{M}=T_{W}+T_{O}-1. \end{array}$$

A simulated acoustic image including a square target of SNR = 10 dB with a size of *T*_o_ = 12 was built. The size of the calculation window was selected as *T*_w_ = 15, and *T*_M_ = 26 could be obtained from equation (4). A theoretical template of the target in the local skewness space was established according to equation (1).

**Figure 6:**
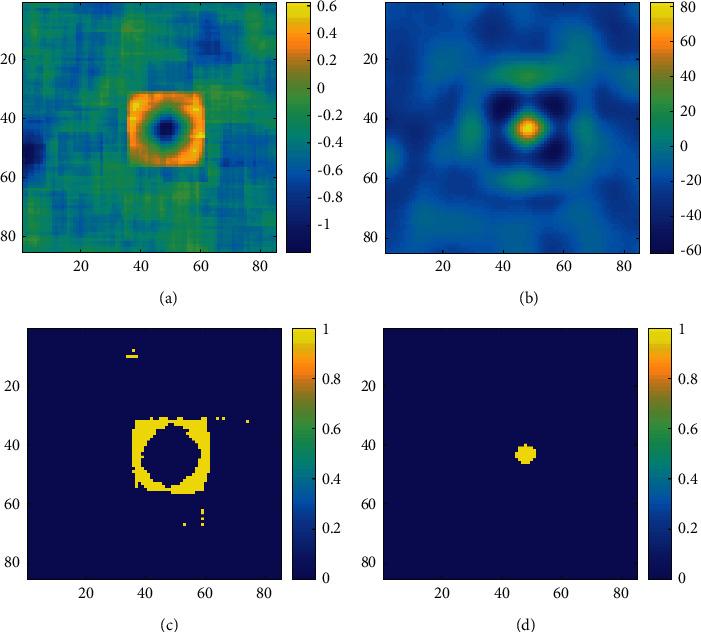
Comparison of detections with and without target focusing. (a) Local skewness without target focusing. (b) Local skewness with target focusing. (c) Detection results without target focusing. (d) Detection results with target focusing.

The local skewness image is shown in Figure 6(a). It is clear that the target in the local skewness image is a special large cube structure when choosing a larger computing window. By correlating the local skewness image with the theoretical template of the target, the result is shown in Figure 6(b). The highlight region formed after focus processing represents the target, and its maximum value is located at the center of the original sound image. SC-CFAR is used for ROI detection in Figures 6(a) and 6(b). In Figure 6(c), without target focusing, the target presents a hollow rectangular shape and there are a few false alarms. In Figure 6(d), the actual appearance and position of target can be easily detected after target focusing.

### 3.3. Uncertain Target Theory Template

In the application, the calculation window size is defined by the researcher and the potential target size and number are unknown. In this case, the theoretical template of the target in the local skewness space cannot be determined. To solve this problem, a theoretical template with an uncertain target size can be designed, which is composed of multiple target theoretical templates with different sizes. The weight coefficient obeys the Gaussian distribution, the typical template size is taken as the mean value, and the uncertainty of the size is taken as the mean square error.

**Figure 7:**
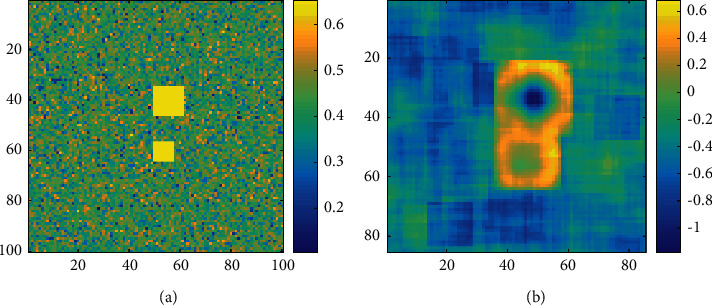
Underwater scene simulation with two targets: (a) simulation acoustic image and (b) local skewness image.

A simulated acoustic image containing two square targets with SNR = 10 dB and sizes of *T*_o1_ = 12 and *T*_o2_ = 6, respectively, is established. The original acoustic image is shown in Figure 7(a), and the gap between the two targets is 10 pixels. The size of the calculation window was selected as *T*_w_ = 15, and the local skewness image is obtained in Figure 7(b) when the target position is close; the overlap of the calculation windows causes the special cubes to be mixed together, which makes the subsequent target detection extremely difficult.

**Figure 8:**
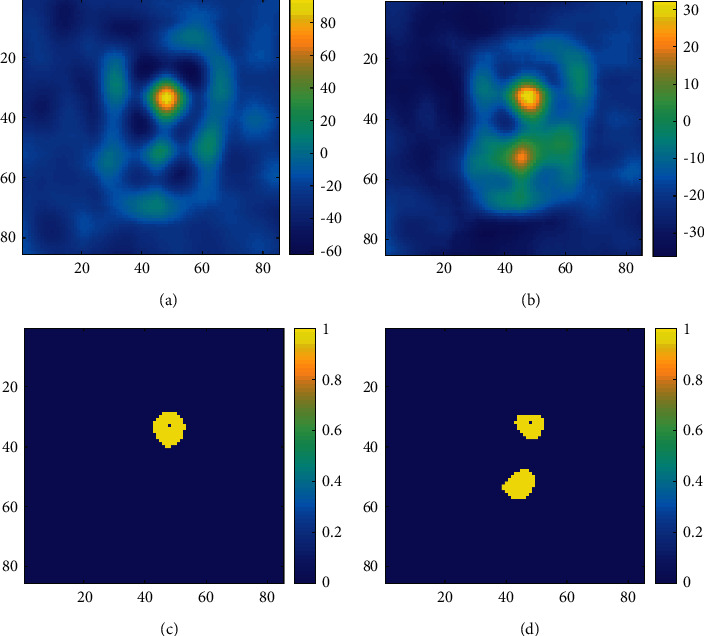
Comparison of focusing with different templates. (a) Focusing with a certain target template. (b) Focusing with an uncertain target template. (c) Detection results with a certain template. (d) Detection results with an uncertain template.

Using the certain target theoretical template and selecting the template size *T*_M_ = 26, the local skewness image with target focusing is shown in Figure 8(a). Only the large one with *T*_o1_ = 12 can be observed, and the small one with *T*_o2_ = 6 is submerged in the background. In this case, if the target detection algorithm is executed directly, smaller targets will be missed. Design an uncertain target theoretical template with the size *T*_M_ = [20–28], and the result after target focusing is shown in Figure 8(b). Two targets can fully be observed, both of which are higher in magnitude than the surrounding background. The results of SC-CFAR detection are shown in Figures 8(c) and 8(d). Only the larger size target can be obtained with a certain target template, while both targets with different sizes and close positions can be clearly detected with an uncertain target template. It is concluded that the uncertain template exhibits better focusing effect and stronger robustness than the certain template, when the target size is unknown or multiple targets exist.

## 4. Experiments

### 4.1. Configuration

**Figure 9:**
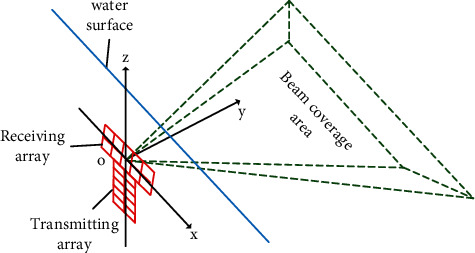
Diagram of fixed-point head-up sonar layout.

The operating frequency of the sonar system used in the experiment is 300 kHz, the sampling frequency is 58 kHz, the receiving array has 65 elements, the number of beams is 256, and the beam coverage is 150° × 1.5°. The multibeam sonar system is located 2 m above the water surface by lifting the rod. The receiving array and the transmitting array are placed along the *X*-axis and *Z*-axis, and the two forms a T-type perpendicular to the *Z*-axis. As shown in Figure 9, the sonar system is fixed and the beam sector is parallel to the water surface. The underwater field of view under this layout can be called fixed-point head-up view.

### 4.2. Evaluation

Experimental evaluation focuses on two aspects: (i) target representation in space; (ii) ROI detection performance. Calculate the target SNR of the original image and the local HOS image by equation (2), and evaluate the target representation in the space by comparison. The detection performance is evaluated by the detection rate *P*_d_ and the observed false alarm rate *P*_fa_, which are defined as

(5) $$\begin{array}{c} P_{d}=\frac{N_{d}}{N_{o}},\\ P_{\text{fa}}=\frac{N_{\text{fa}}}{N_{t}-N_{o}}, \end{array}$$

where *N*_t_ is the sum of pixels of the acoustic image, *N*_o_ is the sum of pixels belonging to the target, *N*_fa_ is the sum of pixels observed as false alarms, and *N*_d_ is the sum of pixels observed as the target.

The proposed method is compared with other methods in terms of ROI detection performance. The method description and parameter setting are shown in Table 1. Method-I directly performs segmentation in the acoustic image, and the threshold range is 0–1, which is set to 0.99. Both Method-II and Method-III carry out target reconstruction in the local HOS space and then perform SC-CFAR. The window is selected as *T*_w_ = 12, and the false alarm rate is set to an equivalent 0.01. Method-II uses a certain theoretical template with a target size *T*_o_ = 9, while Method-III uses an uncertain template with a target range *T*_o_ = [6∼12].

### 4.3. Results and Analysis

The indoor pool experiment is designed to simulate the underwater scene in which the sonar system is fixed in the safe area near the port to monitor the approaching threat target. A large amount of actual data including real targets was collected, and the acoustic image sequences were generated by water imaging.

**Figure 10:**
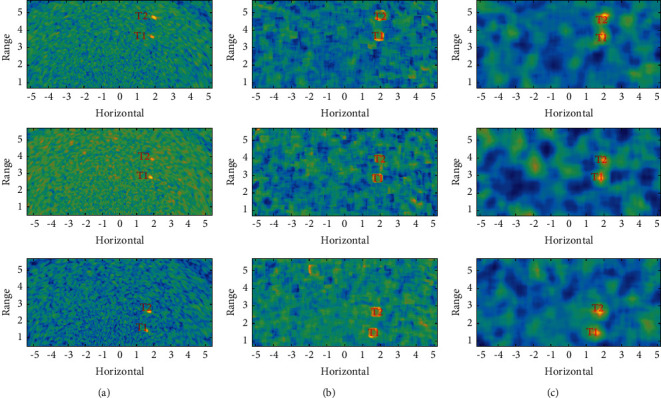
Acoustic image sequence I: (a) original acoustic image, (b) local HOS image, and (c) target reconstruction using uncertain template.

Acoustic image sequence I contains 33 frames of 261 × 541 acoustic images with a resolution of 0.02 × 0.02 m^2^. It describes 5.2 × 10.8 m^2^ water scene parallel to the water surface, in which two targets marked T1 and T2 move along the direction of the track simultaneously. The typical original acoustic image is shown in Figure 10(a), the local HOS image is shown in Figure 10(b), and target reconstruction using an uncertain template is shown in Figure 10(c). The size of the target is small and the echo intensity is close to the background, which makes it difficult to identify the target in the original image. In the local HOS image, the target forms a unique square structure, which shows a clearer original appearance after the target reconstruction.

**Figure 11:**
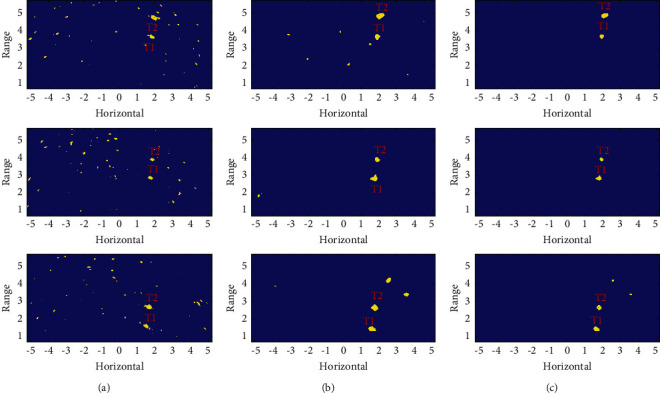
ROI detection on acoustic image sequence I: (a) results with Method-I, (b) results with Method-II, and (c) results with Method-III.

The three methods described in Table 1 are used to perform ROI detection.  Figure 11(a) shows the detection result with Method-I. A large number of false alarms are detected along with the targets. Figures 11(b) and 11(c) show Method-II and Method-III, respectively, and both have achieved better detection results. Using a certain template, the area of the target is slightly larger, while using an uncertain template, the target is closer to the actual appearance and the false alarm rate is lower.

The detection statistics of the image sequence I are shown in Table 2, which lists SNR, *P*_d_, and P_fa_ of the above three images and the average of the entire image sequence. From the original image to the local HOS space, the average SNR of the target is improved by 30.33%. Comparing Method-I and Method-II, Method-III achieved the highest *P*_d_ = 91.35% and the lowest false alarm rate *P*_fa_ = 0.03%.

**Figure 12:**
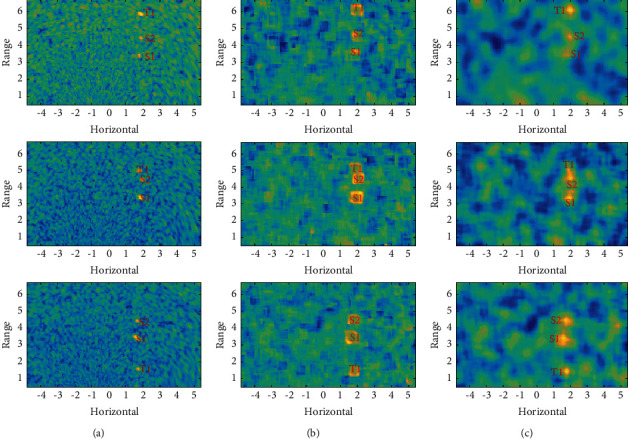
Acoustic image sequence II: (a) original acoustic image, (b) local HOS image, and (c) target reconstruction using an uncertain template.

Acoustic image sequence II contains 48 frames of 125 × 205 acoustic images with a resolution of 0.05 × 0.05 m^2^. It describes a water scene of 6.2 × 10.2 m^2^ parallel to the water surface, containing two groups of targets, one set of relatively stationary targets marked S1 and S2 and another set of moving targets marked T1. The typical original images shown in Figure 12(a) have problems such as large background fluctuations, and the moving target is too close to the stationary target. The proposed method can form a highly discriminative cube structure shown in Figure 12(b) and achieve focus at the target location shown in Figure 12(c).

**Figure 13:**
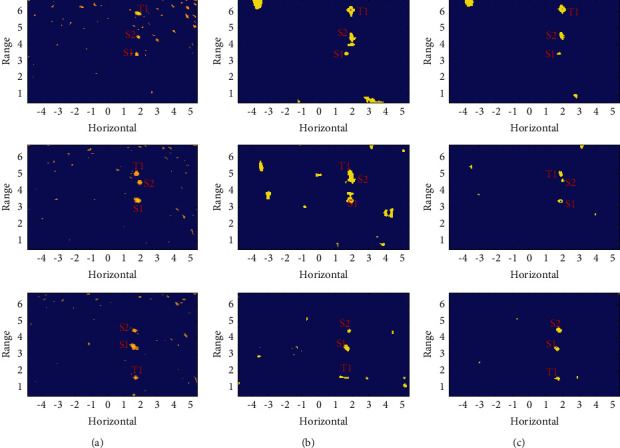
ROI detection on acoustic image sequence II: (a) results with Method-I, (b) results with Method-II, and (c) results with Method-III.

The detection results with Method-I shown in Figure 13(a) still have a high false alarm rate.  Figure 13(b) shows the detection result with Method-II and there are many blocky highlight areas, corresponding to false targets. Method-III can fully detect both stationary and moving targets shown in Figure 13(c), and the false alarm rate is also acceptable.

The detection statistics of the image sequence II are shown in Table 3, and the content displayed is similar to Table 2. Converting to the local HOS space, the average SNR of the target is only improved by 12.20%, but the proposed method still achieves *P*_d_ = 76.87% and the lowest false alarm rate *P*_fa_ = 0.13%.

In summary, the qualitative comparison of the target representation and detection results is given in Figures 10–13 and the corresponding quantitative results are in Tables 2 and 3. The performance of the proposed method is better than that of the traditional methods in terms of *P*_d_ and *P*_fa_. Moreover, the target reconstruction restores the cube structure in local HOS to its original appearance, and the uncertain template is used to achieve better focusing effect than the certain template. The target area has a slight but negligible offset, mainly due to the sliding window. Further improvements can be optimized through window location estimation.

## 5. Conclusion

This paper studies target perception in local HOS space, which is difficult to interpret in an original acoustic image. The main conclusions are as follows:

1. When the original acoustic image is mapped to the local skewness space, the target presents a special cube structure and the SNR is enhanced. Therefore, the target with low SNR is easier to identify in the local skewness space.
2. The cube structure formed by the target in the local skewness space will cause the problem of target positioning. The focus processing can restore the actual appearance of the target as much as possible and determine the position of the target. When the target size and number are unknown, using the uncertain target template can achieve a better effect.
3. An underwater target perception architecture based on layered processing mechanism is proposed and verified by experiments with multiple sets of real data. Experimental results show that compared with the traditional method, the proposed method has a higher detection rate and a lower false alarm rate.

Future research will promote the application of a layered target perception architecture to threat target tracking in warning areas such as wharf, port, and nearshore.

## Figures and Tables

**Table 1:** Method description and parameters setting.

Method	Execution object	Detection algorithm		Theoretical template	
		Algorithm	Parameter	Window size	Target size
I	Acoustic image	Segmentation	Threshold = 0.99	—	—
II	Local HOS space	SC-CFAR	*P*_fa_= 0.01	12	9
III	Local HOS space				6–12

**Table 2:** Detection statistics on the image sequence I.

	SNR		*P*_d_			*P*_fa_		
	Original	HOS	Method-I (%)	Method-II (%)	Method-III (%)	Method-I (%)	Method-II (%)	Method-III (%)
Image1	37.34	45.34	10.93	75.58	96.23	0.63	0.12	0.00
Image2	29.42	44.42	5.06	90.45	100.00	0.67	0.03	0.00
Image3	36.35	44.68	19.64	61.30	84.28	0.57	0.23	0.05
Average	35.05	45.68	14.05	70.94	91.35	0.61	0.17	0.03

Image1, Image2, and Image3 correspond to the three rows of images from the top to bottom in Figures 10 and 11, respectively.

**Table 3:** Detection statistics on the image sequence II.

	SNR		*P*_d_			*P*_fa_		
	Original	HOS	Method-I (%)	Method-II (%)	Method-III (%)	Method-I (%)	Method-II (%)	Method-III (%)
Image1	33.33	38.02	12.84	35.20	52.85	0.88	1.05	0.41
Image2	36.88	40.23	22.18	57.41	87.84	0.79	0.30	0.04
Image3	38.71	41.25	26.46	41.67	79.78	0.75	0.36	0.08
Average	37.37	41.93	26.33	52.69	76.87	0.75	0.55	0.13

Image1, Image2, and Image3 correspond to the three rows of images from the top to bottom in Figures 12 and 13, respectively.I

## Data Availability

The experimental data were collected from the indoor pool of Harbin Engineering University, Harbin, China.
